# *Tadehagi triquetrum* aqueous extract ameliorates diabetic kidney disease through mitigating epithelial senescence via the PTEN/AKT/mTOR signaling pathway

**DOI:** 10.1186/s13020-026-01378-0

**Published:** 2026-03-31

**Authors:** Li Li, Liying Xue, Chu Xue, Ankang Tan, Guicong Chen, Chao Zhang

**Affiliations:** 1 College of Fundamental Medicine, Hainan Vocational University of Science and Technology, Haikou, 571126 China; 2 Hainan Vocational University, Haikou, 570216 China; 3 School of Traditional Chinese Pharmacy, China Pharmaceutical University, Nanjing, 211198 China

**Keywords:** *Tadehagi triquetrum*, Diabetic kidney disease, Cell senescence, PTEN/AKT/mTOR pathway

## Abstract

**Background:**

*Tadehagi triquetrum*, a well-known medicinal plant widely consumed in Asia, possesses hypoglycemic properties. However, its precise impact in managing diabetic complications remains insufficiently understood. This study aims to investigate the protective effects and underlying mechanisms of *T. triquetrum* aqueous extract (TAE) on diabetic kidney disease (DKD).

**Methods:**

The db/db mice were administered TAE at doses of 0.1, 0.2, and 0.4 g/kg for 10 weeks to assess its renoprotective effect. Senescence-associated β-galactosidase (SA-β-Gal) staining, Western blotting, and RT-qPCR were employed to assess the anti-senescence effects of TAE both in vivo and in vitro. RNA-seq was performed to uncover the molecular mechanisms through which TAE ameliorates tubular senescence. The involvement of PTEN/AKT/mTOR pathway was validated using shRNA-mediated gene knockdown in vitro and a pharmacological inhibitor of PTEN in vivo. UPLC-MS/MS analysis and molecular docking were used to identify active components targeting PTEN in TAE.

**Results:**

TAE administration significantly improved renal function and attenuated histopathological damage in db/db mice. Additionally, TAE effectively suppressed cellular senescence in both in vivo and in vitro models, as evidenced by reduced SA-β-Gal-positive area, decreased levels of senescence-associated secretory phenotype, and downregulated expression of senescence-related proteins (P53, P21, and P16). RNA-seq analysis revealed that the anti-senescence effects of TAE were associated with the modulation of the mTOR signaling pathway. Validation experiments confirmed that TAE alleviates epithelial senescence by inhibiting PTEN-mediated activation of mTOR phosphorylation. UPLC-MS/MS analysis and molecular docking identified Hesperidin, Quercitrin, and Carnosol as major bioactive compounds in TAE with high binding affinity for PTEN.

**Conclusions:**

In conclusion, our findings demonstrate that TAE exerts protective effects against epithelial senescence in DKD by modulating the PTEN/AKT/mTOR signaling, highlighting its potential as a novel therapeutic approach for the management of DKD.

**Graphical Abstract:**

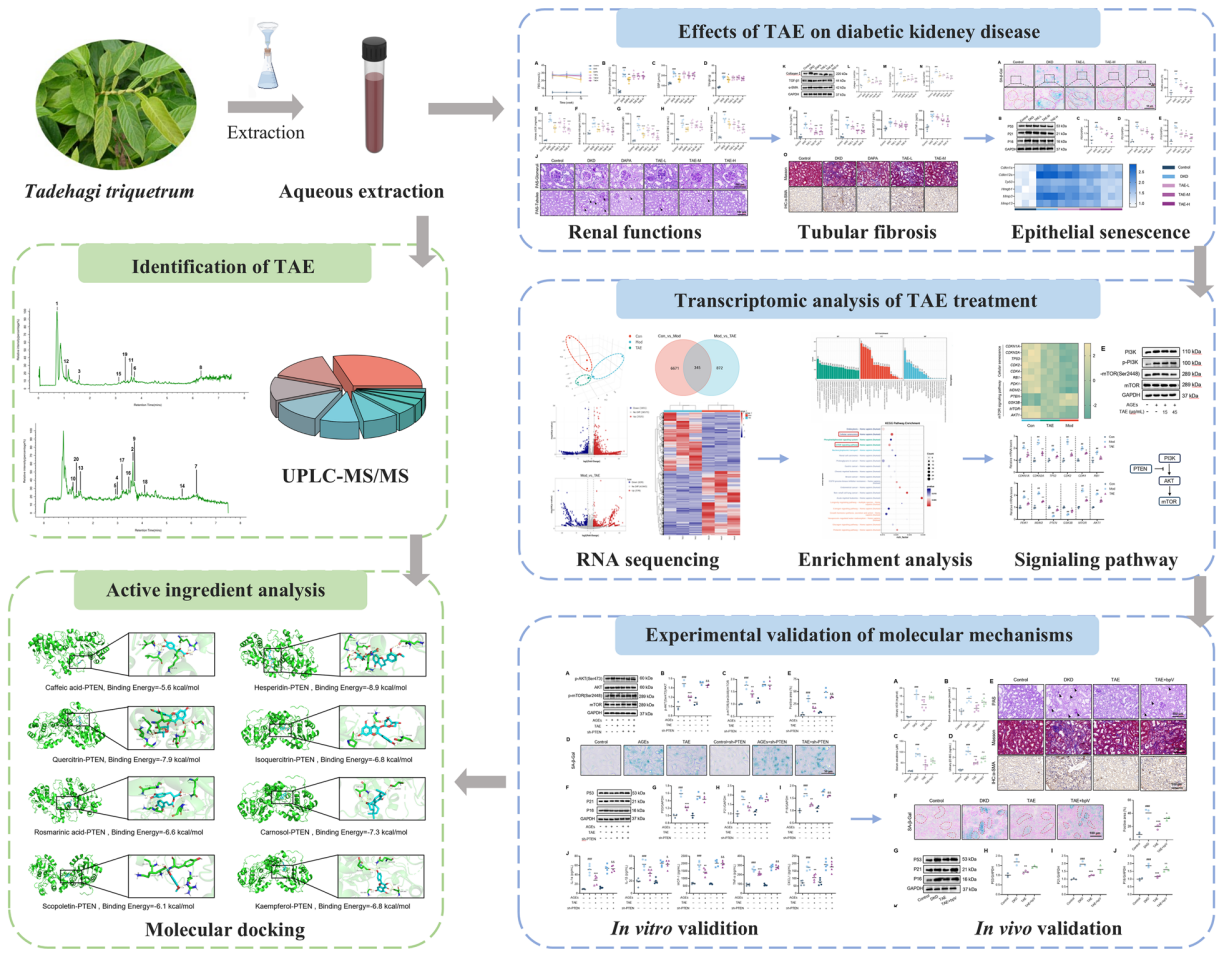

**Supplementary Information:**

The online version contains supplementary material available at 10.1186/s13020-026-01378-0.

## Introduction

Diabetic kidney disease (DKD), characterized by the presence of albuminuria and a low estimated glomerular filtration rate (eGFR), is a leading cause of end-stage renal disease (ESRD) and imposes a significant societal burden as the population ages[8, 10]. Clinical trials have demonstrated that existing treatments, including hypoglycemic agents, sodium-glucose cotransporter protein 2 (SGLT2) inhibitors, and renin–angiotensin–aldosterone system (RAAS) blockers, exhibit limited efficacy in halting the progression of DKD to ESRD [9, 17]. Consequently, the development of novel therapeutic strategies for DKD remains a critical unmet need. Previous studies have shown that elevated urinary β2-microglobulin levels in type 2 diabetic patients occur before overt glomerular damage [40], suggesting that targeting tubular injury may be a promising approach for the early prevention and management of DKD.

Cellular senescence, classically defined as a stable cell cycle arrest, is accompanied by a complex senescence-associated secretory phenotype (SASP) [31]. Renal tubular epithelial cell (TEC) senescence has been extensively documented as a critical cellular event in the progression of early kidney injury [22, 37]. Renal TEC senescence not only inhibits renal regeneration and repair processes but also promotes the transition to chronic kidney disease via a SASP [29]. Clinical studies have confirmed the presence of increased cellular senescence in DKD, as evidenced by elevated Senescence-associated β-Galactosidase-positive areas and P16 protein expression in type 2 diabetic nephropathy biopsies compared with normal age-matched control tissues [32]. Notably, several investigations have demonstrated that targeting renal tubular P21 expression can reverse hyperglycemic memory-induced renal TEC senescence in DKD patients, thereby delaying disease progression [3, 30]. Therefore, protecting renal TECs from senescence represents an important novel strategy for treating early-stage DKD.

The mTOR signaling pathway plays a critical role in regulating cellular metabolism, autophagy, and the cell cycle, thereby influencing cellular senescence [18, 28]. Inhibition of mTOR signaling abnormal activation has been extensively documented to effectively alleviate cellular senescence across various diseases [2, 19, 36]. For instance, C-anthocyanidins mitigate the senescence of mesenchymal stem cells through ZDHHC5-mediated autophagy via regulating the PI3K/AKT/mTOR pathway [12]. Additionally, Punicalagin alleviates cigarette smoke extract-induced bronchial epithelial cell senescence through the PAR2/mTOR pathway [33]. On the other hand, aberrant of the mTOR signaling pathway have been implicated in the progression of DKD. Specifically, upregulation of DNMT1 in diabetic immune cells induces abnormal cytosine methylation of upstream regulators of mTOR, leading to pathological activation of the mTOR pathway and subsequent inflammation in the diabetic kidney [23]. However, it is noteworthy that no studies have yet specifically addressed the impact of the mTOR signaling pathway on renal TEC senescence in the context of DKD.

*Tadehagi triquetrum* (L.) H. Ohashi is a well-known medicinal plant widely distributed across Asia. Traditionally, this plant has been utilized for tea production and in folk medicine for the management of diabetes [27]. Previous studies have demonstrated that *T. triquetrum* significantly enhances glucose uptake and consumption in HepG2 cells [39], indicating its potential application in managing diabetes and its associated complications. Hence, the potential therapeutic effects of *T. triquetrum* on DKD need further investigate. In this study, the renal protective and anti-senescence effect of *T. triquetrum* aqueous extract (TAE) was assessed both in vivo and in vitro, with its underlying mechanisms investigated through RNA-seq analysis and validation experiments. Our findings revealed that TAE protects diabetic kidney from injury and fibrosis through mitigating epithelial senescence via the PTEN/AKT/mTOR signaling pathway. We anticipate that this study will provide a potentially safe treatment strategy for patients with DKD.

## Materials and methods

### Preparation of *T. triquetrum*

The leaf of *T. triquetrum* [Fabaceae], named according to Linnaean binomial nomenclature, was collected in Baisha Li Autonomous County of Hainan Province, People’s Republic of China, and identified by Prof. Liping Huang from Hainan Vocational University. The voucher specimen (No. L20210033) was deposited in the Department of Fundamental Medicine of Hainan Vocational University. The air-dried leaf of *T. triquetrum* was soaked in double-distilled water (ddH_2_O) for 30 min and extracted twice by boiling in ddH_2_O (using a solvent-to-solid ratio of 10:1) for 1 h each. The consequent extracting solution was combined and yielded *T. triquetrum* aqueous extract (TAE).

### UPLC-MS/MS analysis

#### Liquid chromatography conditions

The liquid chromatography analysis was performed on a Vanquish UHPLC System (Thermo Fisher Scientific, USA) equipped with an ACQUITY UPLC HSS T3 column (2.1 × 100 mm, 1.8 µm) (Waters, Milford, MA, USA). The column was maintained at 40 ℃. The flow rate and injection volume were set at 0.3 mL/min and 2 μL, respectively. For LC-ESI ( +)-MS analysis, the mobile phases consisted of (A) 0.1% (*v/v*) formic acid aqueous solution and (B) 0.1% formic acid in acetonitrile. For LC-ESI (-)-MS analysis, the mobile phases consisted of (A) 5 mmol/L ammonium formate aqueous solution and (B) acetonitrile. The gradient was set as follows: 0–1 min, 10% B; 1–5 min, 10%-98% B; 5–6.5 min, 98% B; 6.5–6.6 min, 98%-10% B; 6.6–8 min, 10% B. The flow rate of mobile phases was set at 0.3 mL/min.

#### Mass spectrum conditions

Mass spectrometric detection of metabolites was performed on a coupled Q Exactive Focus Orbitrap mass spectrometer (Thermo Fisher Scientific, USA) with an ESI ion source. Simultaneous full scan and ddMS2 modes were used for precursor and fragment ions acquisition. The spray voltage was 3.50 kV and −2.50 kV for ESI ( +) and ESI (−), respectively. The capillary temperature was set at 325 ℃. The ranges of scan were set at m/z 100–1000 for MS1 and 50–1000 for MS2.

#### Data analysis

The raw data were initially converted into mzXML format using MSConvert from the ProteoWizard software package (v3.0.8789) and subsequently processed with R XCMS (v3.12.0) for feature detection, retention time correction, and alignment. Ions were identified based on accurate mass and MS/MS data by matching against a database including METLIN, HMDB, LipidMap, KEGG, ChEBI, and an in-house metabolite database build by Panomix Biomedical Tech Co., Ltd. (Suzhou, China).

### Animals and drug treatment

Animal studies were conducted following protocols approved by the Ethics Committee for animal research of China Pharmaceutical University (Approval AEWC-20230913-2). Male db/db mice (8 weeks old) and age-matched db/m mice were purchased from GemPharmatech (Nanjing, China). All mice were maintained under controlled humidity and temperature conditions in the Center for Experimental Animals at China Pharmaceutical University with access to food and water ad libitum. One week after acclimatization, the animals were randomly divided into six groups (*n* = 6 per group): Control; DKD; TAE-L (0.1 g/kg); TAE-M (0.2 g/kg); TAE-H (0.4 g/kg); DAPA (1 mg/kg). The db/db mice were administered TAE or DAPA via oral gavage, with a volume of 0.1 mL per 20 g of body weight. Age-matched db/m mice were given equal volumes of saline by gavage and used as controls. In the validation experiments, db/db mice received intraperitoneal administration of bpV(HOpic) at a dose of 1 mg/kg/d to inhibit PTEN, in combined with TAE-H treatment. After 10 weeks of drug treatment, the animals were euthanized by intraperitoneal injection of pentobarbital sodium (30 mg/kg), followed by decapitation. No mouse mortality was observed throughout this study; blood, urine, and renal samples were collected for further analysis.

### Biochemical analysis

The fasting blood glucose (FBG) levels were evaluated from the tail vein blood of mice fasting overnight with a blood glucose meter (Roche Diagnostics). The levels of glycated serum protein (GSP; Elabscience, Wuhan, China), urinary albumin (Elabscience, Wuhan, China), blood urea nitrogen (BUN; Elabscience, Wuhan, China), creatinine, and β2-microglobulin (β2-MG; Elabscience, Wuhan, China) in serum or urine were measured by commercial kits following the manufacturer’s protocol.

### Pathological analysis

Mouse kidney tissues were fixed with 4% paraformaldehyde (PFA; Solarbio, Beijing, China), embedded in paraffin, sectioned, and stained with Periodic Acid-Schiff (PAS) and Masson solution (Beyotime, Beijing, China) according to the manufacturer’s protocol. For immunohistochemistry (IHC) staining, the expression of alpha-smooth muscle actin (α-SMA) in kidney tissues was evaluated following previously published methods.

### Senescence-associated β-galactosidase (SA-β-Gal) staining

Mouse kidney tissues were rapidly frozen with liquid nitrogen, embedded in optimal cutting-temperature compound (OST, Sakura, USA), and sectioned. The tissue sections and cultured HK2 cells were stained with SA-β-Gal staining solution (Solarbio, Beijing, China) according to the manufacturer’s description.

The SA-β-gal staining cells were observed under a microscope. Images of cultured cells were taken under 20 × objective lens. Images of frozen sections were taken under 20 × or 40 × objective lens. The SA-β-Gal-positive area within each field was quantified using ImageJ software. The mean positive area (%) from randomly five fields was calculated as the result for each sample.

### Western blot analysis

Proteins were extracted from the collected kidney tissue and cells using RIPA lysis buffer supplemented with PMSF on ice. Protein content was determined using the Enhanced BCA Protein Assay Kit (Beyotime, Beijing, China) according to the manufacturer’s instructions. Equal protein samples were subjected to SDS-PAGE, transferred onto nitrocellulose (NC) membrane, blocked with 5% BSA for 2 h, and incubated with primary antibodies as follows at 4 °C overnight. Next, the membranes were incubated with a horseradish peroxidase (HRP)-conjugated secondary antibody and visualized using a commercial enhanced chemiluminescence kit. Grey values were quantified using Image J software and normalized to expressions of GAPDH as an internal standard. Antibodies used in this study are listed in Table S1.

### Detection of SASP

The levels of SASP factors, including interleukin-1α (IL-1α), interleukin-1β (IL-1β), monocyte chemoattractant protein-1 (MCP-1), tumor necrosis factor-α (TNF-α), and C-X-C motif chemokine ligand 1 (CXCL1), in serum and cell supernatants were quantified using commercial kits (Jiangsu Meimian Industrial Co., Ltd, China) according to the manufacturer’s instructions.

### Preparation of advanced glycation end-products

Advanced glycation end-products (AGEs) were prepared according to previously published methods [6, 24]. In brief, bovine serum albumin (BSA; Solarbio, Beijing, China) was incubated with methylglyoxal (Macklin, Shanghai, China) at 37 ^◦^C in the dark for 14 days. Unbound methylglyoxal was subsequently removed through dialysis. AGEs were identified based on fluorescence measurements (excitation, 370 nm; emission, 440 nm).

### Cell culture and treatments

The human kidney 2 cells (HK2; ATCC) were cultured in DMEM/F12 basic medium (KeyGen, Nanjing, China) supplemented with 10% fetal bovine serum (FBS, Gibco, USA) at 37 ℃ and 5% CO_2_. HK2 cells were treated with AGEs (4 mg/mL) for 48 h to induce cellular senescence in vitro. TAE was administered at concentrations of 15 and 45 μg/mL in cell experiments.

### RNA sequencing analysis

Total RNA was extracted separately from HK2 cells, AGEs-induced HK2 cells, and AGEs-induced HK2 cells with TAE treatment using RNA isolater Total RNA Extraction Reagent (Vazyme, Nanjing, China), separately. The EdgeR algorithm was applied to filter differentially expressed genes (DEGs) through |log₂FC|> 1 and adjusted P < 0.05. Cluster analysis and principal component analysis (PCA) were performed based on DEGs. The DEGs underwent functional enrichment analysis utilizing the DAVID database (https://david.ncifcrf.gov/home.jsp) for Gene Ontology (GO) functional annotation and the Kyoto Encyclopedia of Genes and Genomes (KEGG) pathway annotation. The raw RNA-seq data were deposited in NCBI Sequence Read Archive and are available under the BioProject accession number PRJNA1420181.

### Cell transfection

Short hairpin RNAs targeting PTEN (sh*PTEN*-1/2/3) and their negative control (shNC) were designed and synthesized by KeyGen (Nanjing, China). HK2 cells were transfected with the aforementioned plasmids using Lipofectamine 3000 reagent (Invitrogen, USA) strictly according to the manufacturer’s instructions. Forty-eight hours post-transfection, the transfection efficacy was evaluated by detecting PTEN expression via western blot and RT-qPCR analyses.

### Real-time PCR (RT-qPCR)

Total RNA was extracted using RNA extraction reagent and reverse-transcribed into cDNA with All-in-One First-Strand Synthesis Master Mix (Best Enzymes, Lianyungang, China). qPCR was performed using Taq-HS SYBR Green qPCR Premix (Best Enzymes, Lianyungang, China) on a QuantStudio 6 Flex system. The 2^−ΔΔCt^ method was applied to calculate relative gene expression levels.

### Statistical analysis

The Kolmogorov–Smirnov test was used to assess the normality of the data. For data with normal distributions, one-way ANOVA with Tukey’s post-hoc test was utilized to evaluate differences among more than two groups and adjust for multiple comparisons. All data were presented as Mean ± standard error of mean (SEM), and a *P* value < 0.05 was considered statistically significant.

## Results

### TAE protects the kidney from injury and fibrosis in DKD mice

**Fig. 1:**
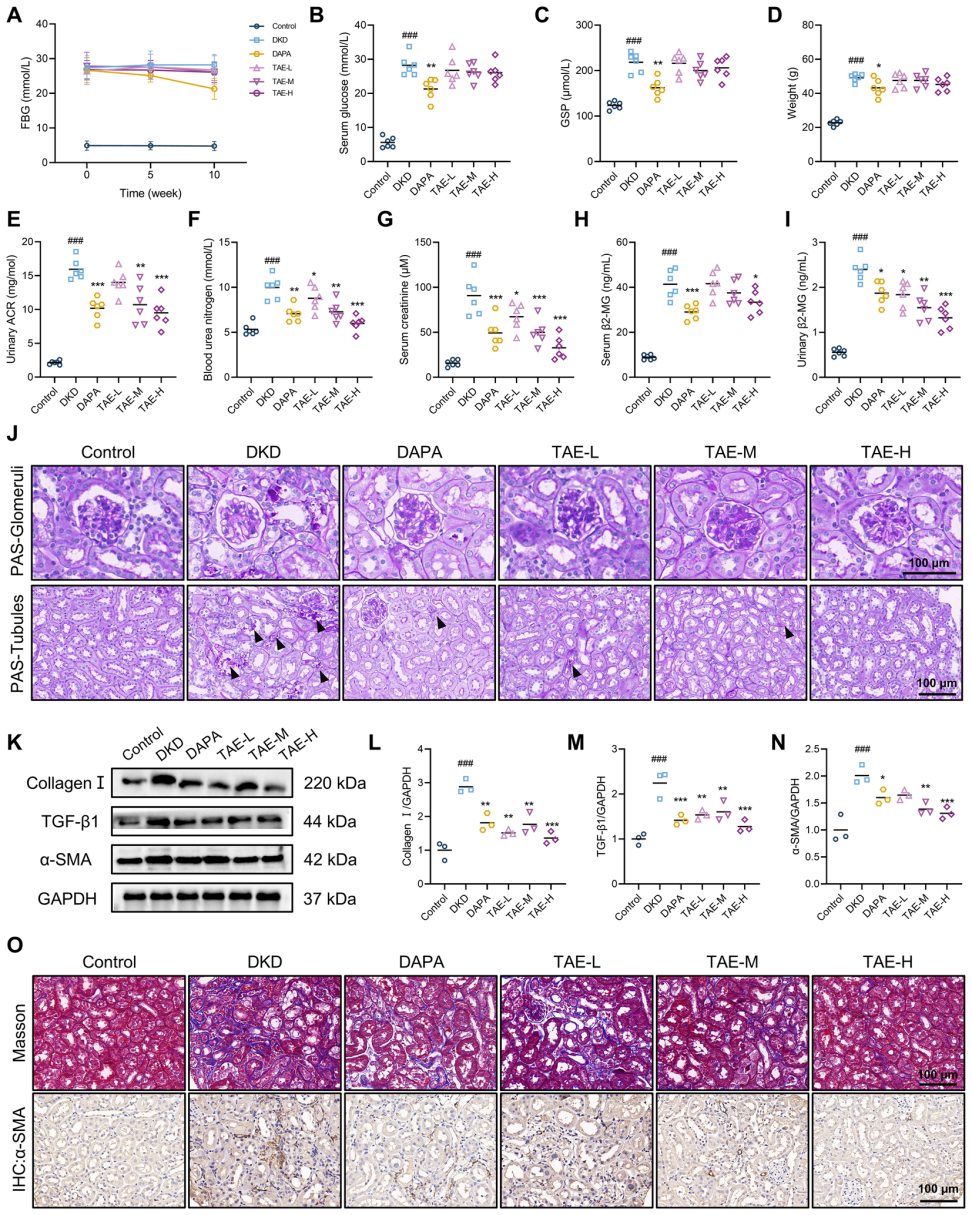
TAE protects kidney from injury and fibrosis in DKD mice. **A** Fasting blood glucose levels during the administration period. **B** The serum glucose levels. **C** Glycated serum protein levels. **D** Body weight. **E–I** The urinary ACR (**E**), blood urea nitrogen (**F**), serum creatinine (**G**), serum β2-MG (H), and urinary β2-MG (**I**) levels were measured using commercial kits at the end of drug administration (*n* = 6 per group). **J** PAS staining in kidney tissues (*n* = 3 per group). Scale bar = 100 μm. **K–N** Fibrosis-related proteins were detected using Western blot analysis (*n* = 3 per group). Representative images (**K**). Quantification of Collagen Ⅰ expression (**L**), TGF-β expression (**M**), α-SMA expression (**N**). **O** Masson staining and immunohistochemistry staining of α-SMA of kidney tissues (*n* = 3 per group). Scale bar = 100 μm. Data are presented as the means ± SEM. ^###^*P* < 0.001 *vs.* Control group; *P < 0.05, **P < 0.01, ***P < 0.001 *vs.* DKD group

**Fig. 2:**
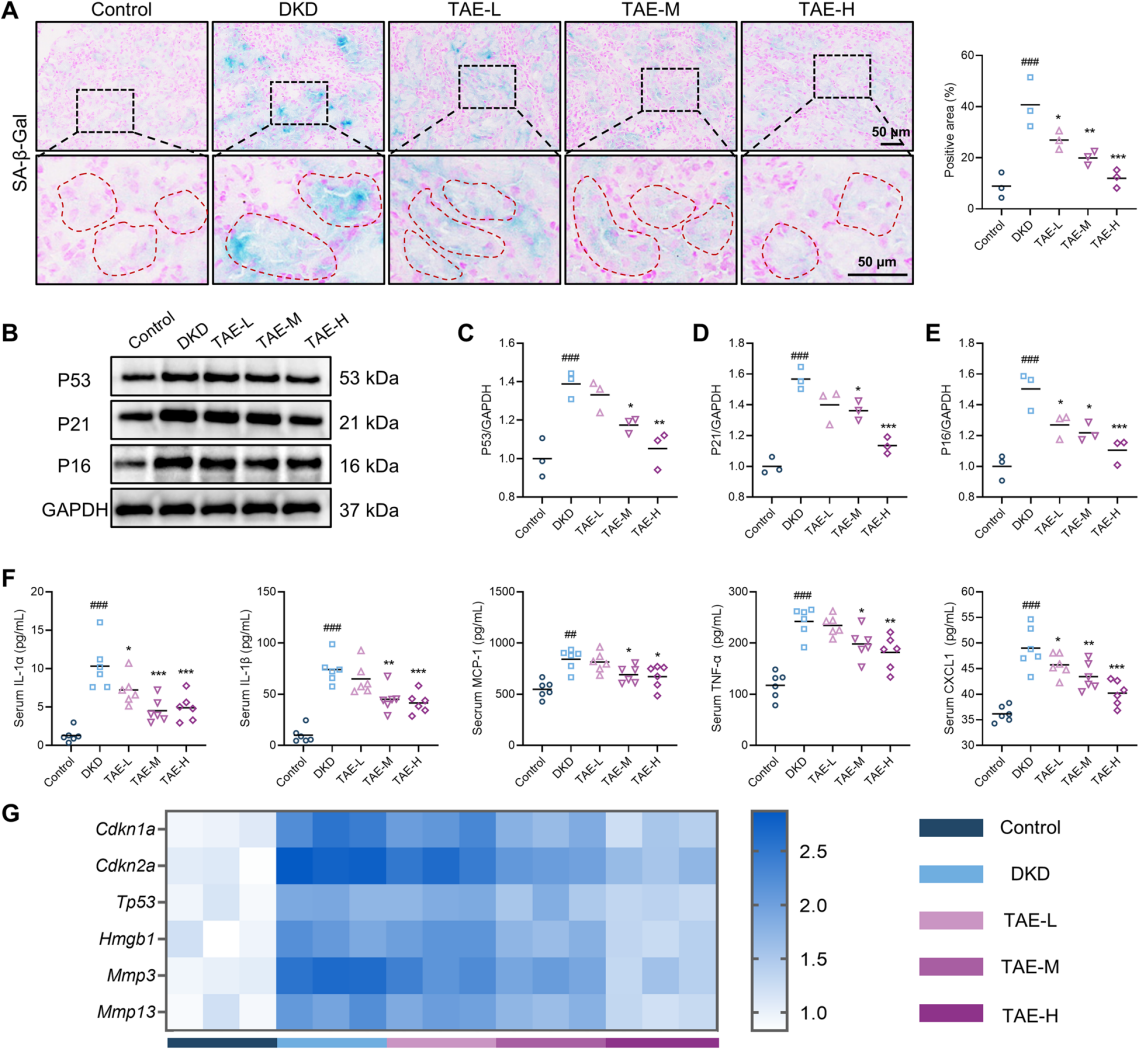
TAE alleviates renal tubular senescence in DKD mice.** A** Representative images and quantification of SA-β-Gal staining in kidney tissue (*n* = 3 per group). Scale bar = 50 μm. **B**–**E** Cell senescence-related proteins (P53, P21, and P16) were detected by Western blot analysis (*n* = 3 per group). Representative images (**B**). Quantification of P53 expression (**C**), P21 expression (**D**), P16 expression (**E**). **F** The levels of IL-1α, IL-1β, MCP-1, TNF-α, and CXCL1 in serum were tested using ELISA (*n* = 6 per group). **G** Cell senescence-related genes (*Cdkn1a*, *Cdkn2a*, *Tp53*, *Hmgb1*, *Mmp3*, and *Mmp13*) were assessed using RT-PCR (*n* = 3 per group). Data are presented as the means ± SEM. ^##^*P* < 0.01, ^###^*P* < 0.001 *vs.* Control group; *P < 0.05, **P < 0.01, ***P < 0.001 *vs.* DKD group

To investigate the therapeutic effects of TAE on DKD, db/db mice were treated with TAE for 10 weeks, with dapagliflozin (DAPA) serving as the positive control. As shown in Fig. 1A, B, db/db mice exhibited significantly elevated fasting blood glucose (FBG) levels compared to db/m mice. Notably, TAE administration did not lead to a statistically significant decrease in FBG levels or serum glucose levels. Similarly, TAE treatment failed to reduce the increased GSP levels and body weight in db/db mice (Fig. 1C, D). The db/db mice also exhibited significantly increased water and food intake, but TAE treatment did not modulate these changes (Figure S1A-B). However, urinary albumin-to-creatinine ratio (ACR) was reduced in db/db mice following TAE or dapagliflozin treatment compared to the DKD group (Fig. 2E). Consistently, TAE markedly decreased the levels of serum BUN and serum creatinine, indicating a potent renoprotective effect (Fig. 2F, G). To further assess glomerular and tubular injury, serum and urinary β2-MG levels were measured, respectively. As shown in Fig. 1H, I, TAE showed minimal improvement in serum β2-MG levels but significantly reduced urinary β2-MG levels, with the high-dose group outperforming DAPA. Furthermore, PAS staining revealed that TAE treatment effectively alleviated tubular damage by reducing loss of tubular epithelial cells and protein casts, although no significant improvement was observed in glomerular morphology (Fig. 1J). Next, the effect of TAE on renal fibrosis was evaluated. Western blot analysis demonstrated that TAE significantly decreased the expression of collagen Ⅰ, TGF-β1, and α-SMA compared to untreated db/db mice (Fig. 1K–N). These findings were further confirmed by Masson staining and IHC analysis of α-SMA, which showed reduced collagen deposition and α-SMA expression after 10 weeks of TAE administration (Fig. 1O). Collectively, these results indicate that TAE ameliorates kidney injury and fibrosis in db/db mice.

### TAE alleviates renal tubular senescence in DKD mice

Previous studies have demonstrated that targeting renal tubular P21 expression in renal tubules can reverse hyperglycemic memory-induced tubular epithelial cell senescence in DKD patients, thereby delaying disease progression. In this study, SA-β-Gal staining was conducted to evaluate the in vivo effects of TAE on tubular senescence. The results revealed a significant increase in SA-β-Gal-positive areas in the renal tubules of db/db mice compared to age-matched controls, which was markedly attenuated by TAE treatment (Fig. 2A). Subsequently, Western blot analysis was performed to detect cellular senescence-associated proteins, demonstrating that TAE treatment significantly attenuated the upregulation of P53, P21, and P16 protein expression in the kidney of DKD mice (Fig. 2B–E). As illustrated in Fig. 2F, SASP levels were assessed using ELISA. The findings showed that DKD mice exhibited a significant increase in IL-1α, IL-1β, MCP-1, TNF-α, and CXCL1 levels in serum compared to controls; however, TAE treatment effectively suppressed these increases. Moreover, RT-PCR analysis indicated that db/db mice treated with TAE showed significantly lower mRNA levels of *Cdkn1a*, *Cdkn2a*, *Tp53*, *Hmgb1*, *Mmp3*, and *Mmp13* of kidney tissue compared to untreated db/db mice (Fig. 2G). In summary, these results confirm that TAE alleviates renal tubular senescence in db/db mice.

### TAE improves epithelial senescence of AGEs-induced HK2 cells

**Fig. 3:**
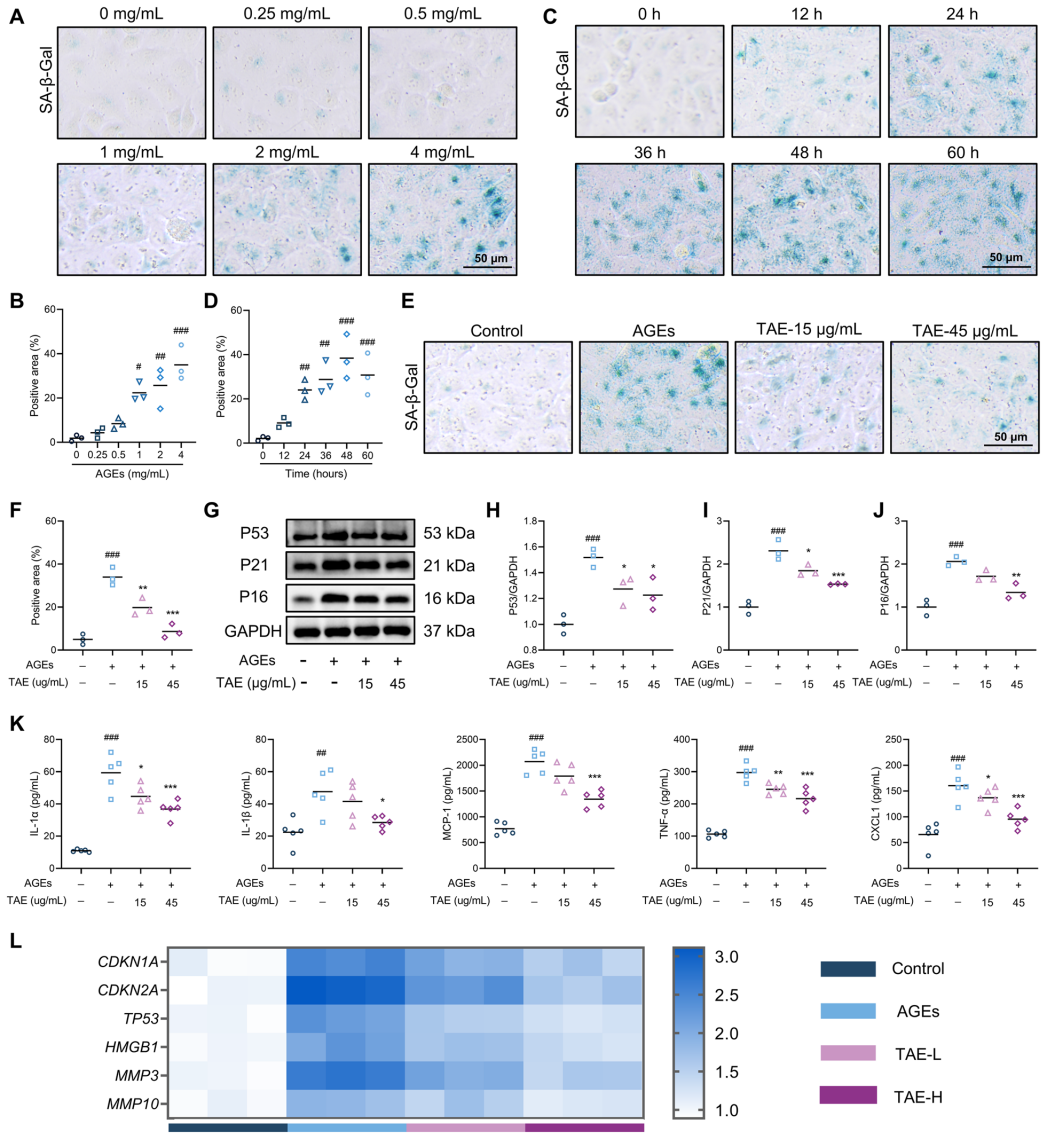
TAE improves epithelial senescence of AGEs-induced HK2 cells.** A**, **B** Representative images and quantification of SA-β-Gal staining in HK2 cells exposed to different concentrations of AGEs (0.25, 0.5, 1, 2, and 4 mg/mL) for 24 h (*n* = 3 per group). Scale bar = 50 μm. **C**, **D** Representative images and quantification of SA-β-Gal staining in HK2 cells exposed to 4 mg/mL for different time (12, 24, 36, 48, and 60 h), (*n* = 3 per group). Scale bar = 50 μm. **E, F** Representative images and quantification of SA-β-Gal staining in AGEs-induced HK2 cells treated with or without TAE (*n* = 3 per group). Scale bar = 50 μm. **G**-**J** Cell senescence-related proteins (P53, P21, and P16) were detected by Western blot analysis (*n* = 3 per group). Representative images (**G**). Quantification of P53 expression (**H**), P21 expression (**I**), P16 expression (**J**). **K** The levels of IL-1α, IL-1β, MCP-1, TNF-α, and CXCL1 in AGEs-induced HK2 cells supernatant were tested using ELISA (*n* = 6 per group). **L** Cell senescence-related genes (*CDKN1A*, *CDKN2A*, *TP53*, *HMGB1*, *MMP3*, and *MMP13*) were assessed using RT-PCR (*n* = 3 per group). Data are presented as the means ± SEM. ^#^*P* < 0.05, ^##^*P* < 0.01, ^###^*P* < 0.001 *vs.* Con group; *P < 0.05, **P < 0.01, ***P < 0.001 *vs.* Mod group

In vitro, HK2 cells were cultured in the presence of AGEs to mimic the senescence conditions of renal TECs in DKD. Initially, HK2 cells were exposed to various concentrations of AGEs (0.25, 0.5, 1, 2, and 4 mg/mL) for 24 h. As shown in Fig. 3A, B, SA-β-Gal staining results indicated that AGEs at concentrations of ≥ 1 mg/mL significantly increased the SA-β-Gal-positive areas of HK2 cells compared to controls. Based on the maximal induction of senescence, 4 mg/mL AGEs was selected for subsequent experiments. Next, HK2 cells were incubated with 4 mg/mL AGEs for 12, 24, 36, 48, and 60 h, respectively. The results revealed that 48 h of AGEs exposure induced the highest level of senescence, as evidenced by the greatest increase in SA-β-Gal-positive areas (Fig. 3C, D). Therefore, in subsequent experiments, HK2 cells were incubated with 4 mg/mL AGEs for 48 h.

No significant reduction in cell viability was observed following treatment with concentrations up to 45 μg/mL. Thus, 15 and 45 μg/mL TAE were selected for follow-up experiments (Figure S2A). As shown in Fig. 3E, F, TAE treatment significantly reduced the SA-β-Gal-positive areas in AGEs-induced HK2 cells, as demonstrated by SA-β-Gal staining. Consistently, western blot analysis revealed that TAE markedly attenuated the AGEs-induced elevation of P53, P21, and P16 protein levels in HK2 cells (Fig. 3G–J). ELISA outcomes further showed that TAE significantly downregulated the secretion of IL-1α, IL-1β, MCP-1, TNF-α, and CXCL1 in the supernatants of AGEs-induced HK2 cells (Fig. 3K). Additionally, RT-PCR analysis indicated that TAE treatment significantly reduced the mRNA levels of *CDKN1A*, *CDKN2A*, *TP53*, *HMGB1*, *MMP3*, and *MMP13* in HK2 cells, which were elevated by AGEs exposure (Fig. 3L). In short, our data indicate that TAE improves the epithelial senescence of AGEs-induced HK2 cells.

### TAE rescues epithelial senescence is associated with mTOR signaling pathway

**Fig. 4:**
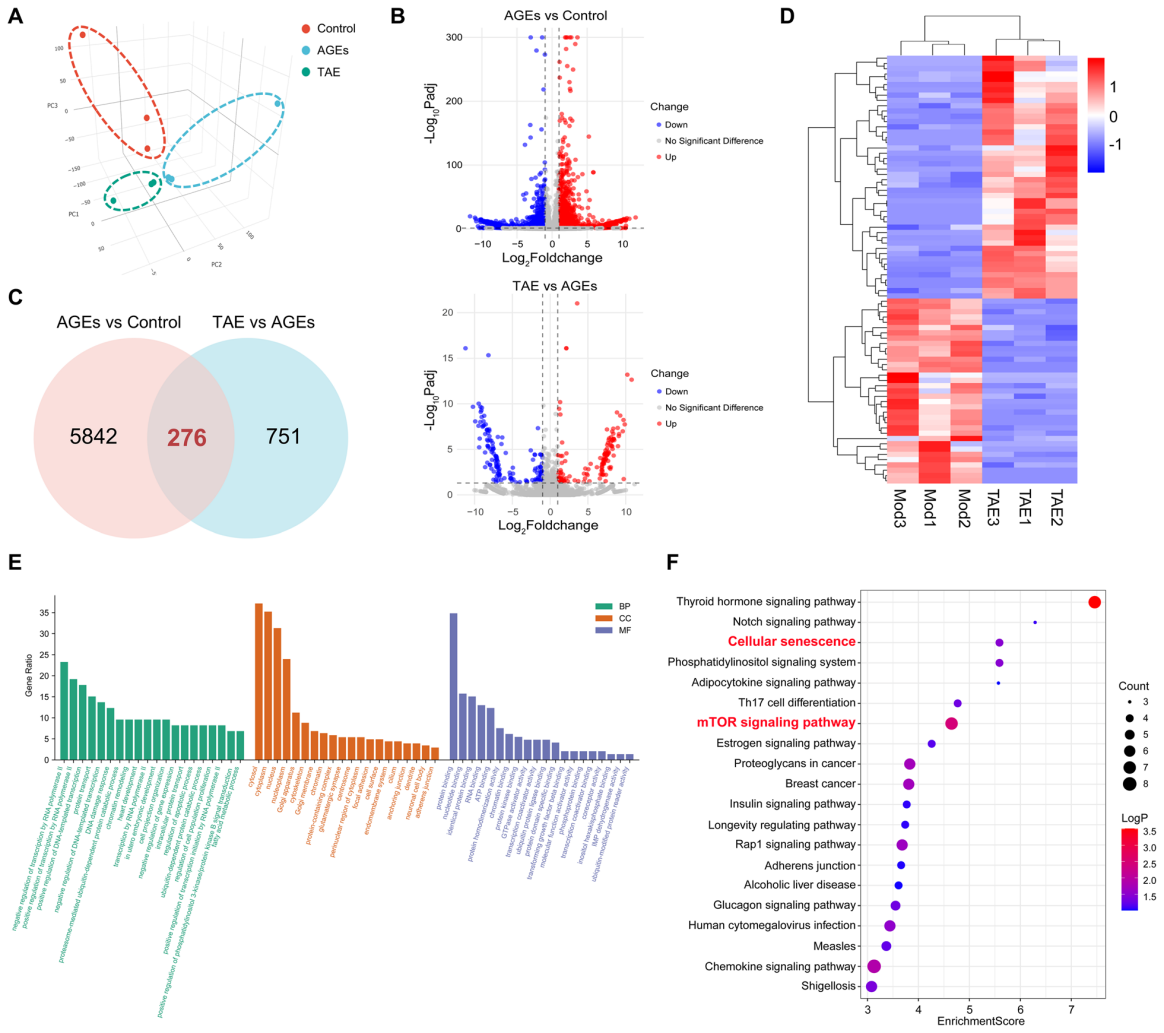
TAE rescues epithelial senescence is associated with mTOR signaling pathway. **A**–**F** The HK2 cells (Con group), AGEs (4 mg/mL)-induced HK2 cells (AGEs group), and AGEs (4 mg/mL)-induced HK2 cells treated with TAE (45 mg/mL, TAE group) were teasted using RNA-seq (*n* = 3 per group). Principal component analysis of DEGs (**A**). Volcano maps depicting DEGs (**B**). Venn of DEGs (**C**). Heatmap of DEGs between AGEs and TAE groups (**D**). GO enrichment analysis between AGEs and TAE groups (E). KEGG pathway enrichment analysis between AGEs and TAE groups (F)

To explore the underlying mechanism by which TAE improves epithelial senescence under DKD conditions, RNA-seq analysis was conducted here. As shown in Fig. 4A, the principal component analysis (PCA) results demonstrated distinct separation of clusters among the Control, AGEs, and TAE treatment groups, with no overlap observed. a total of 276 commonly differentially expressed genes were identified across these groups. (Fig. 4B, C). Cluster analysis heatmaps revealed a significant difference in gene profiles between the AGEs and TAE treatment groups (Fig. 4D). Subsequently, GO enrichment analysis and KEGG pathway enrichment analysis were performed to investigate the potential mechanism. The “Regulation of cell population proliferation” and other pathways were enriched through GO enrichment analysis (Fig. 4E). In Fig. 4F, KEGG pathway enrichment analysis showed that DEGs between AGEs and TAE treatment groups were enriched in “Cellular senescence” and “mTOR signaling pathway”. In conclusion, these findings demonstrate that TAE inhibits epithelial senescence is associated with mTOR signaling pathway.

### TAE inhibits the activation of mTOR signaling pathway

**Fig. 5:**
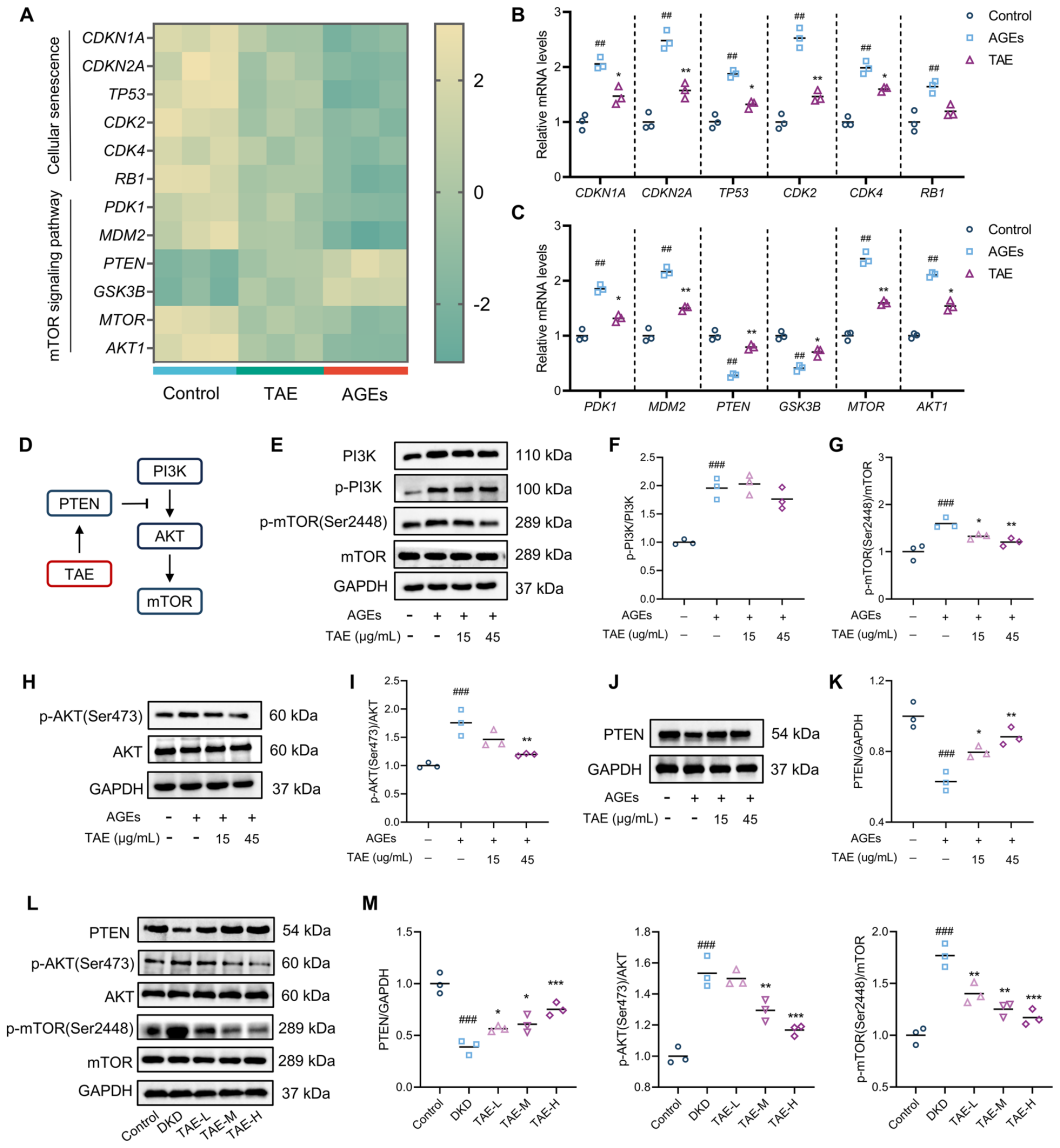
TAE inhibits the activation of mTOR signaling pathway.** A** Heatmap of DEGs in “cellular senescence” and “mTOR signaling pathway” datasets (*n* = 3 per group). **B**, **C** Validation of DEGs using RT-PCR (*n* = 3 per group). **(D)** Key proteins in mTOR signaling pathway. **E–K** Key proteins in mTOR signaling pathway were detected by Western blot analysis (*n* = 3 per group). Representative images (**E**, **H**, and **J**). Quantification of p-PI3K/PI3K expression (**F**), p-mTOR/mTOR expression (**G**), p-AKT/AKT expression (**H**), and PTEN expression (**K**). **L**, **M** Key proteins in mTOR signaling pathway of animals (*n* = 3 per group). Representative images (**L**). Quantification of PTEN expression, p-mTOR/mTOR expression, and p-AKT/AKT expression (**M**). Data are presented as the means ± SEM. ^##^*P* < 0.01, ^###^*P* < 0.001 *vs.* Con or Control group; *P < 0.05, **P < 0.01 *vs.* AGEs or DKD group

To further investigate the mechanism by which TAE regulates mTOR signaling pathway, differentially expressed genes among the Con, AGEs, and TAE groups involved in the “Cellular senescence” and “mTOR signaling pathway” datasets were analyzed. As shown in Fig. 5A, cellular senescence-related genes such as *CDKN1A*, *CDKN2A*, and *TP53*, as well as mTOR signaling pathway-related genes including *MTOR* and *AKT1*, were significantly decreased following TAE treatment compared to the Mod group, which is consisted with our RT-PCR outcomes (Fig. 5B, C). Given the well-documented role of the PI3K/AKT/mTOR signaling pathway in regulating cellular senescence, the expression levels of key proteins in this pathway were measured (Fig. 5D). Western blot analysis revealed that AGEs led to a significant downregulation of PTEN expression and marked upregulation of p-PI3K, p-AKT (Ser473) and p-mTOR (Ser248) levels in HK2 cells. Notably, TAE treatment significantly reversed these alterations, except for p-PI3K (Fig. 5E–K). Moreover, as illustrated in Fig. 5L, M, DKD mice exhibited reduced PTEN expression and increased p-AKT and p-mTOR expression, with no significant differences in total AKT or mTOR expression compared to controls. TAE treatment significantly reversed these alterations, manifested as increased PTEN level and decreased phosphorylation of AKT and mTOR expression. In conclusion, these data suggest that TAE suppresses the activation of mTOR signaling pathway.

### TAE rescues epithelial senescence through regulating the PTEN/AKT/mTOR pathway

**Fig. 6:**
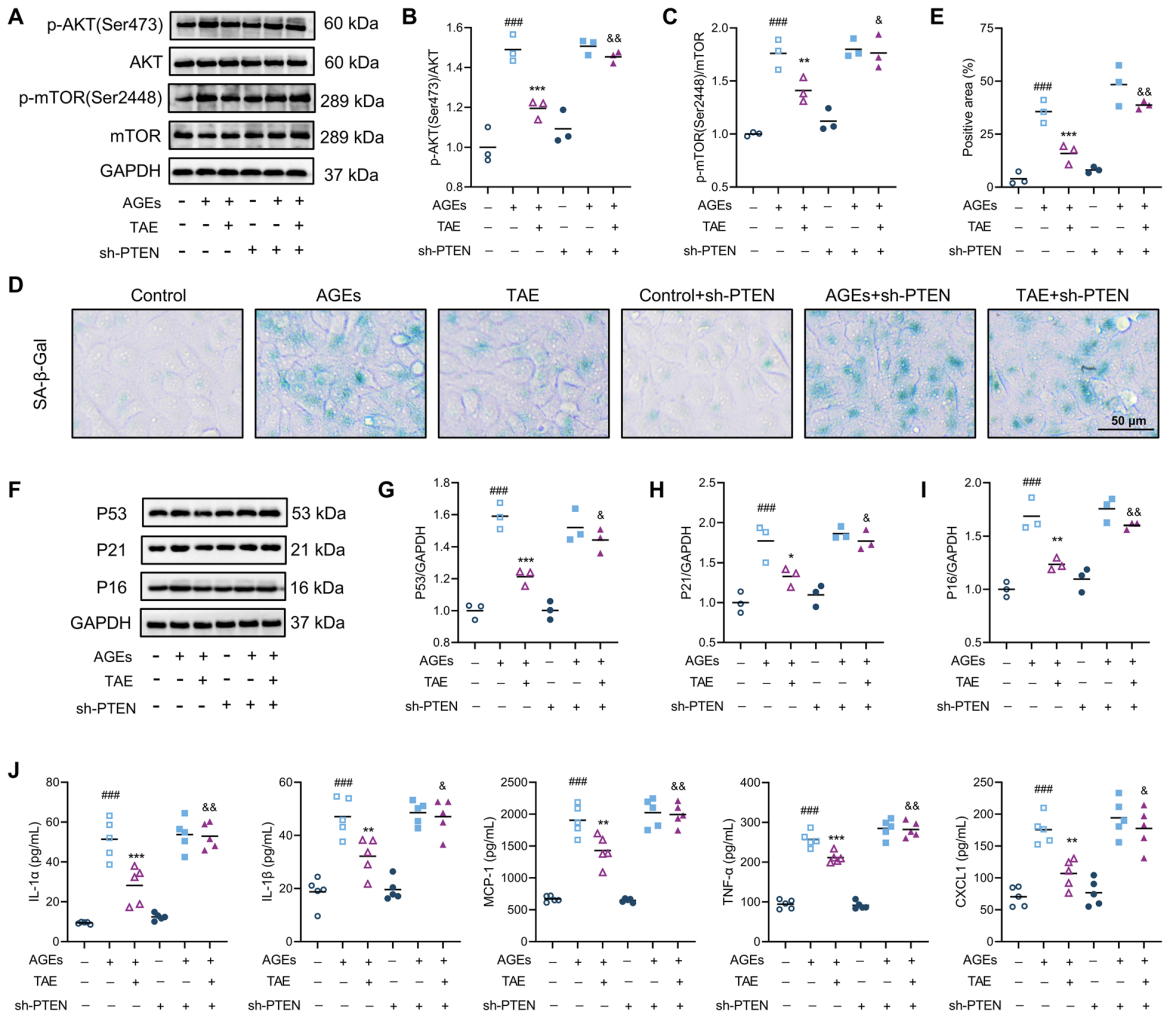
TAE rescues epithelial senescence through regulating the PTEN/AKT/mTOR pathway.** A-C** The related protein levels of mTOR signaling pathway (p-AKT, AKT, mTOR, and p-mTOR) were measured using Western blot analysis (*n* = 3 per group). Representative images (**A**). Quantification of p-AKT/AKT expression (**B**), p-mTOR/mTOR expression (**C**). **D**, **E** Representative images and quantification of SA-β-Gal staining (*n* = 3 per group). Scale bar = 50 μm. **F**, **I** Cell senescence-related proteins (P53, P21, and P16) were detected by Western blot analysis (*n* = 3 per group). Representative images (**F**). Quantification of P53 expression (**G**), P21 expression (**H**), P16 expression (**I**). **J** The levels of IL-1α, IL-1β, MCP-1, TNF-α, and CXCL1 in AGEs-induced HK2 cells supernatant were tested using ELISA (*n* = 6 per group). Data are presented as the means ± SEM. ^###^*P* < 0.001 *vs.* Con group; *P < 0.05, **P < 0.01, ***P < 0.001 *vs.* AGEs group; ^&^*P* < 0.05, ^&&^*P* < 0.01 *vs.* TAE group

Subsequently, sh-PTEN was used in this study to validate the involvement of the PTEN/AKT/mTOR pathway in the anti-senescence effects of TAE. The knockdown efficiency validation indicated that sh-PTEN significantly decreased both mRNA levels and protein expression of PTEN in HK2 cells compared to sh-NC (Figure S2B-C). As shown in Fig. 6A–C, Western blot analysis indicated that PTEN knockdown attenuated the TAE-induced reduction in phosphorylated AKT (Ser473) and phosphorylated mTOR (Ser248) levels. Additionally, SA-β-Gal staining results revealed that PTEN knockdown significantly increased the SA-β-Gal-positive area in AGEs-induced HK2 cells treated with TAE, compared to the TAE group (Fig. 6D, E). Furthermore, Western blot data suggested that the suppressive effects of TAE on senescence-related proteins (P53, P21, and P16) were reversed upon PTEN knockdown (Fig. 6F–I). Consistently, similar reversal was observed in SASP factor secretion, with elevated levels of IL-1α, IL-1β, MCP-1, TNF-α, and CXCL1 detected in the cell supernatant (Fig. 6J). Overall, the above findings confirm that TAE alleviates epithelial cell senescence through modulation of the PTEN/AKT/mTOR signaling pathway.

### TAE alleviates kidney injury by regulating the PTEN/AKT/mTOR pathway

**Fig. 7:**
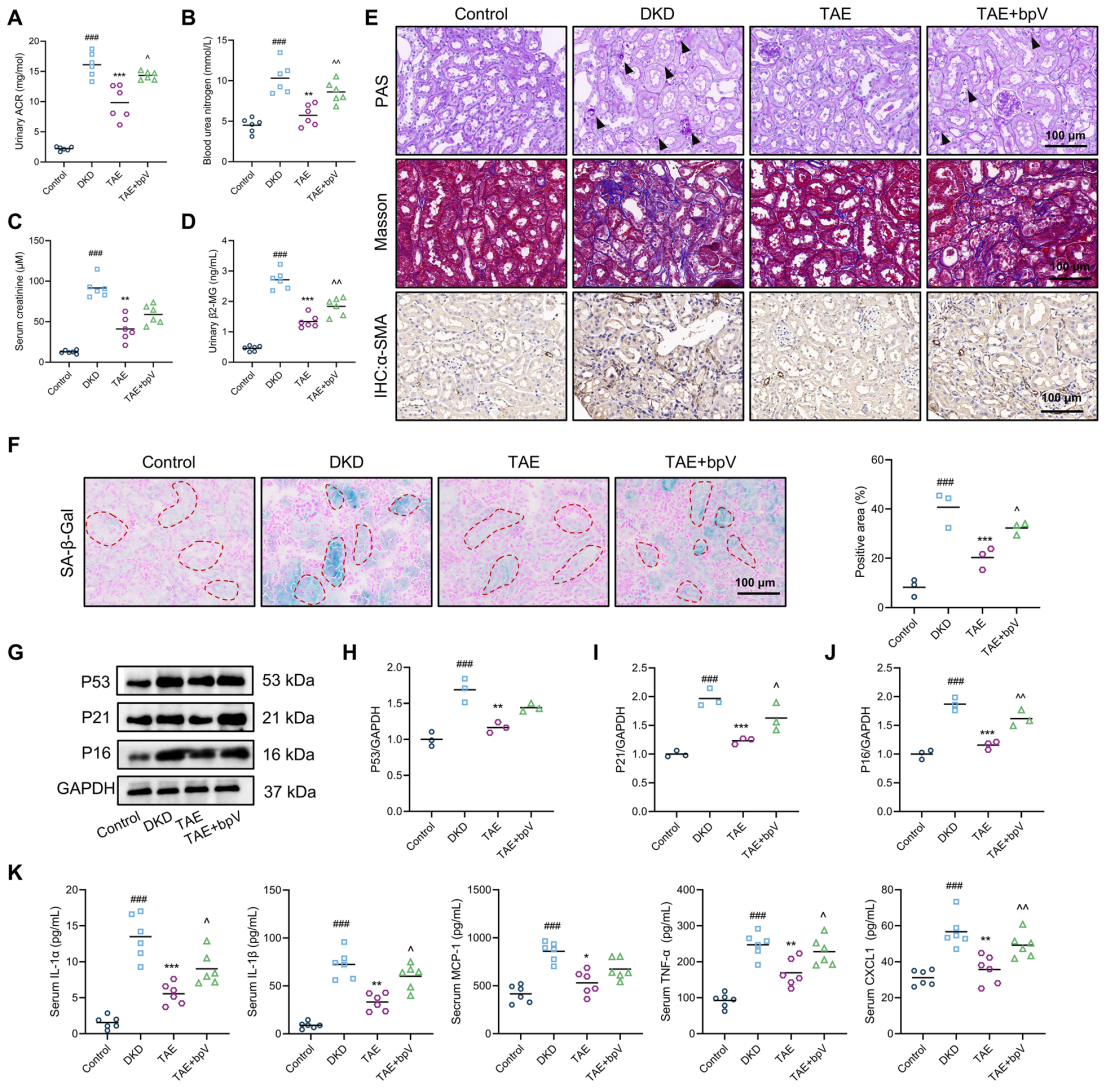
TAE alleviates kidney injury by regulating the PTEN/AKT/mTOR pathway.** A**–**D** The urinary ACR (**A**), blood urea nitrogen (**B**), serum creatinine (**C**), and urinary β2-MG (**D**) levels were measured using commercial kits at the end of drug administration (*n* = 6 per group). **E** PAS staining, Masson staining, and immunohistochemistry staining of α-SMA of kidney tissues (*n* = 3 per group). Scale bar = 100 μm. **F** Representative images and quantification of SA-β-Gal staining in kidney tissue (*n* = 3 per group). Scale bar = 100 μm. **G**–**J** Cell senescence-related proteins (P53, P21, and P16) were detected by Western blot analysis (*n* = 3 per group). Representative images (**G**). Quantification of P53 expression (**H**), P21 expression (**I**), P16 expression (**J**). **K** The levels of IL-1α, IL-1β, MCP-1, TNF-α, and CXCL1 in serum were tested using ELISA (*n* = 6 per group). Data are presented as the means ± SEM. ^###^*P* < 0.001 *vs.* Control group; *P < 0.05, **P < 0.01, ***P < 0.001 *vs.* DKD group; ^*P* < 0.05, ^^*P* < 0.01 *vs.* TAE group

To further confirm that the therapeutic effect of TAE in protecting the kidney from injury and fibrosis is mediated through regulation of the PTEN/AKT/mTOR pathway, bpV(HOpic), a potent and selective inhibitor of PTEN, was employed. As shown in Fig. 7A–D, TAE treatment significantly decreased urinary ACR, blood urea nitrogen, serum creatinine, and urinary β2-MG levels of db/db mice. Notably, co-administration of bpV significantly reversed the beneficial effects of TAE, as evidenced by increased levels of urinary ACR, blood urea nitrogen, and urinary β2-MG. Similarly, db/db mice treated with TAE exhibited increased glycogen deposition, collagen area, and α-SMA expression when co-treated with bpV (Fig. 7A–D). Next, epithelial senescence was assessed. SA-β-Gal staining revealed that PTEN inhibition significantly increased the SA-β-Gal-positive area compared to TAE treatment alone (Fig. 7F). In Fig. 7G–J, Western blot analysis showed that mice in the TAE + bpV group had higher protein expression of P53, P21, and P16 than those in the TAE group. Moreover, bpV significantly reversed the suppressive effects of TAE on SASP factor expression, as demonstrated by elevated levels of serum IL-1α, IL-1β, MCP-1, TNF-α, and CXCL1 (Fig. 7K). In short, those findings indicate that TAE alleviates kidney injury through regulating the PTEN/AKT/mTOR pathway.

### Characterization of active components targeting PTEN in TAE

**Fig. 8:**
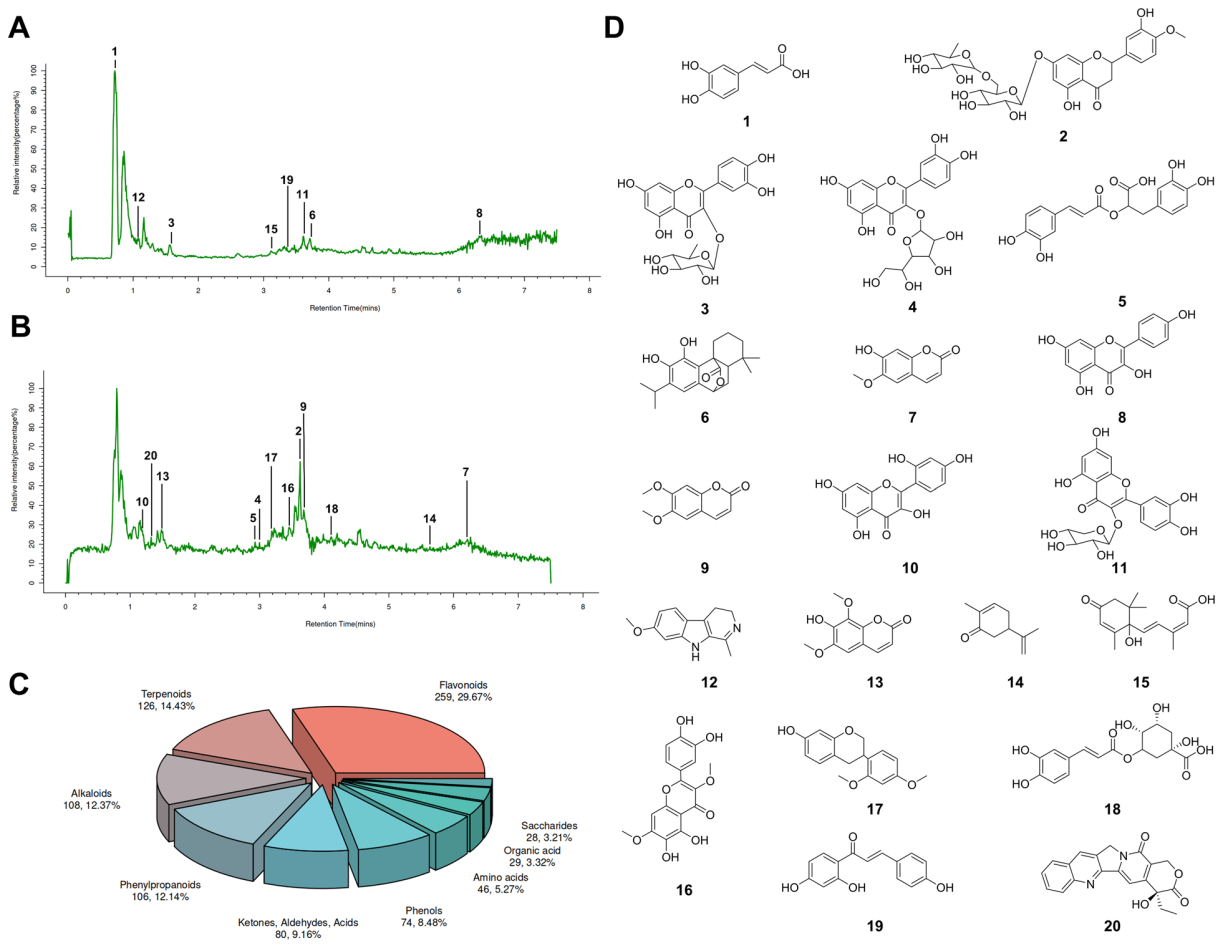
Characterization of chemical components of TAE.** A** The total ion current chromatogram in positive ion mode of TAE. **B** The total ion current chromatogram in negative ion mode of TAE. **C** The proportion and numbers of each classes of chemical components of TAE. **D** The structural formulas of the top 20 compounds in TAE

**Fig. 9:**
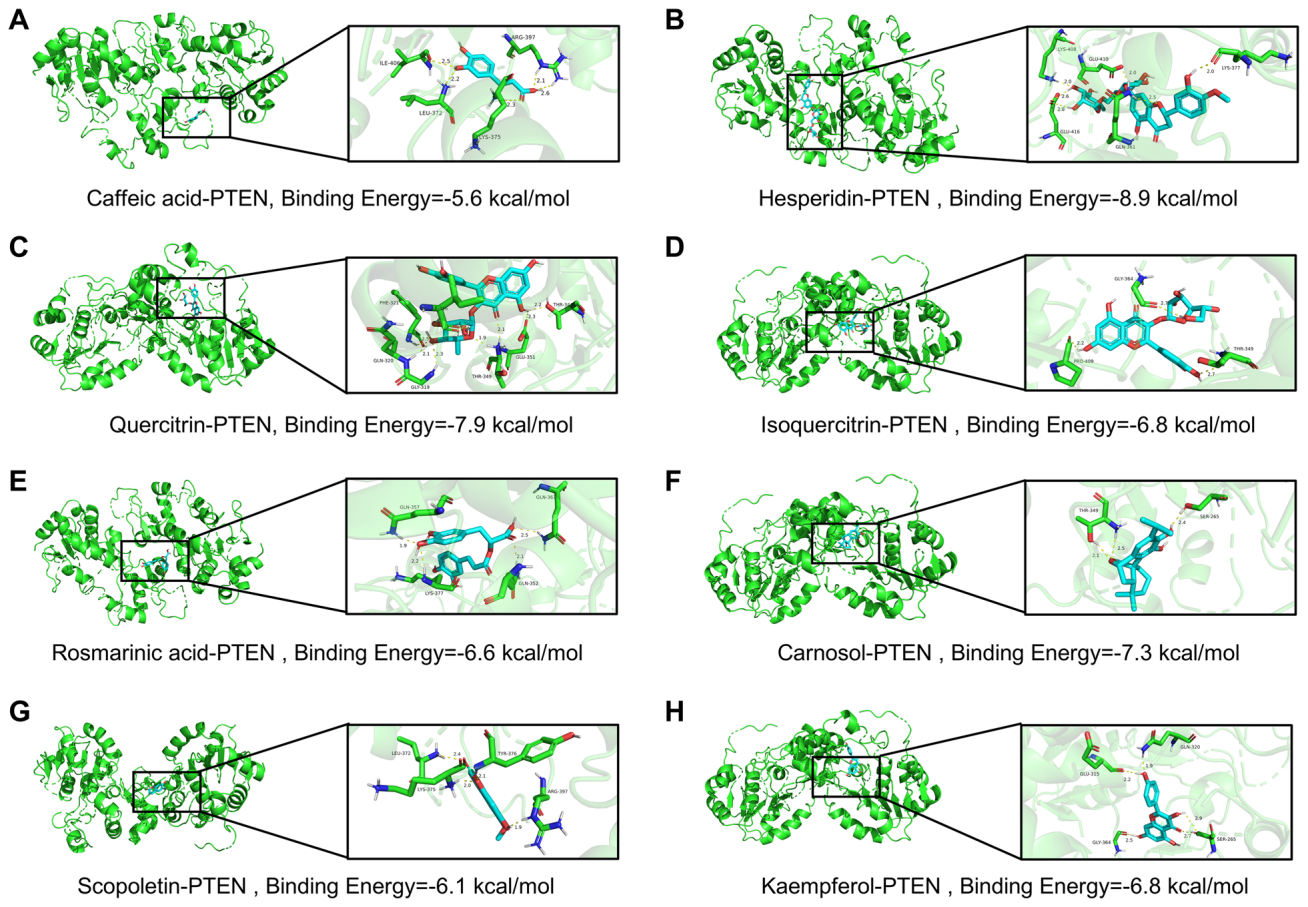
Identification of active ingredients targeting PTEN in TAE. **A**–**H** Molecular docking analysis. **A** Caffeic acid-PTEN. **B** Hesperidin-PTEN. **C** Quercitrin-PTEN. **D** Isoquercitrin-PTEN. **E** Rosmarinic acid-PTEN. **F** Carnosol-PTEN. **G** Scopoletin-PTEN. **H** Kaempferol-PTEN

UPLC-MS/MS analysis was performed to identify the chemical components of TAE, and the total ion flow profiles in both negative and positive ion modes were presented in Fig. 8A, B, respectively. A total of twenty-six classes of chemicals were identified, with flavonoids being the most abundant class, represented by 259 metabolites (Fig. 8C). The top 20 most abundant chemical components in TAE were summarized in Table 1 and Fig. 8D. To identify potential bioactive compounds in TAE that bind to PTEN, molecular docking analysis was performed. As illustrated in Fig. 9A–H, the binding free energy of Hesperidin, Quercitrin, and Carnosol with PTEN are all below−7 kcal/mol, suggesting strong binding affinities. Notably, Hesperidin forms hydrogen bonds with PTEN residues LYS-408, GLU-416, GLU-410, GLN-361, and LYS-377, indicating a stable and specific interaction that warrants further investigation.

**Table 1:** The top 20 abundant chemical components in TAE

Number	Name	Formula	CAS	Exact mass (Da)	Class	Relative intensity (%)	RSD (%)
1	Caffeic acid	C_9_H_8_O_4_	331–39-5	180.042	Phenylpropanoids	1.148 ± 0.015	1.31
2	Hesperidin	C_28_H_34_O_15_	520–26-3	610.190	Flavonoids	0.997 ± 0.027	2.71
3	Quercitrin	C_21_H_20_O_11_	522–12-3	448.100	Flavonoids	0.927 ± 0.023	2.48
4	Isoquercitrin	C_21_H_20_O_12_	21,637–25-2	464.096	Flavonoids	0.791 ± 0.014	1.77
5	Rosmarinic acid	C_18_H_16_O_8_	20,283–92-5	360.085	Phenylpropanoids	0.785 ± 0.027	3.44
6	Carnosol	C_20_H_26_O_4_	5957–80-2	330.183	Terpenoids	0.699 ± 0.038	5.4
7	Scopoletin	C_10_H_8_O_4_	92–61-5	192.042	Phenylpropanoids	0.686 ± 0.034	4.96
8	Kaempferol	C_15_H_10_O_6_	520–18-3	286.048	Flavonoids	0.557 ± 0.022	3.95
9	Scoparone	C_11_H_10_O_4_	120–08-1	206.058	Phenylpropanoids	0.522 ± 0.018	3.45
10	Morin	C_15_H_10_O_7_	480–16-0	302.043	Flavonoids	0.517 ± 0.015	2.90
11	Guaijaverin	C_20_H_18_O_11_	22,255–13-6	434.085	Flavonoids	0.484 ± 0.017	3.51
12	Harmaline	C_13_H_14_N_2_O	304–21-2	214.111	Alkaloids	0.477 ± 0.041	8.59
13	Isofraxidin	C_11_H_10_O_5_	486–21-5	222.053	Phenylpropanoids	0.388 ± 0.034	8.76
14	(r)-carvone	C_10_H_14_O	6485–40-1	150.105	Terpenoids	0.361 ± 0.022	6.09
15	Abscisic acid	C_15_H_20_O_4_	21,293–29-8	264.136	Terpenoids	0.316 ± 0.036	11.39
16	Tomentin	C_17_H_14_O_8_	59,171–23-2	346.069	Flavonoids	0.308 ± 0.015	4.87
17	Sativan	C_17_H_18_O_4_	41,743–86-6	286.121	Flavonoids	0.285 ± 0.027	9.47
18	Chlorogenic acid	C_16_H_18_O_9_	327–97-9	354.091	Phenylpropanoids	0.268 ± 0.039	14.55
19	Isoliquiritigenin	C_15_H_12_O_4_	961–29-5	256.073	Flavonoids	0.243 ± 0.017	7.00
20	Camptothecin	C_20_H_16_N_2_O_4_	7689–03-4	348.111	Alkaloids	0.193 ± 0.019	9.84

## Discussion

In this study, UPLC-MS/MS analysis showed that twenty-six classes of chemicals were identified in TAE. Results from animal experiments demonstrated that TAE significantly rescued the impaired renal function and reduced epithelial senescence in db/db mice. RNA-seq analysis further revealed that the anti-senescence effects of TAE on AGEs-induced HK2 cells were closely associated with the modulation of the mTOR signaling pathway. Validation experiments confirmed that TAE improved epithelial senescence in DKD by regulating the PTEN/AKT/mTOR pathway.

*Tadehagi triquetrum* has been traditionally used in folklore for the management of diabetes mellitus [11]. Previous studies have demonstrated that it possesses multiple biological activities, including hypoglycemia [38], antioxidant [25], and anti-inflammatory properties [26], which are closely associated with the development of diabetes mellitus and its complications [11]. In this study, we demonstrated that TAE effectively improved renal function and attenuated renal fibrosis in animal model of DKD. Notably, TAE treatment did no alter blood glucose and GSP levels of db/db mice, suggesting that its renoprotective effects are independent of glycemic control. Mechanistically, TAE markedly suppressed tubular epithelial cell senescence, a key pathological driver of DKD progression; this suppression was strongly correlated with the observed functional and structural improvements in the kidney. Collectively, these findings suggest that *T. triquetrum* may serve as a valuable resource for the discovery of novel anti-DKD drugs. Nevertheless, it is undeniable that the current scientific research on *T. triquetrum* remains limited, and more studies are needed to provide robust evidence supporting its efficacy. Therefore, our future work will focus on comprehensively investigating the chemical composition of *T. triquetrum* and identifying its anti-senescence active compounds.

Growing evidence has indicated that mitigating renal senescence can effectively delay or inhibit the progression of DKD [3, 7]. Interestingly, current research on renal anti-senescence predominantly focuses on the renal tubules. This may be attributed to the relatively simple structure of the renal tubule, which is composed solely of tubular epithelial cells, in contrast to the intricate architecture of the glomerulus [4, 21]. Moreover, renal tubules appear to be more vulnerable to hyperglycaemic toxicity and ageing processes. Clinically, altered urine composition in diabetic patients, particularly elevated levels of tubular damage markers such as β2-MG, often precedes evident glomerular pathological changes [20]. In this study, our findings demonstrated that TAE significantly alleviated renal TEC senescence both in vivo and in vitro, as evidenced by reduced SA-β-Gal-positive areas, decreased secretion of SASP factors, and downregulated expression of senescence-related proteins. Additionally, Chang et al*.* reported that canagliflozin mitigated diabetic tubulopathy by inhibiting tubular senescence via suppression of hedgehog interacting protein [5]. Collectively, these results underscore the potential of targeting renal tubular senescence as an effective therapeutic strategy for early-stage DKD.

mTOR, as a central regulator of cellular metabolism regulated by AKT, integrates multiple signals associated with cellular senescence. Accumulating evidence demonstrates that modulating the AKT/mTOR signaling pathway effectively alleviates cellular senescence. For instance, C-phycocyanin mitigates the senescence of mesenchymal stem cells by promoting ZDHHC5-mediated autophagy through the PI3K/AKT/mTOR pathway [12]. Oleanolic acid inhibits senescence through IGF-1, affecting the PI3K/AKT/mTOR signaling pathway [34]. PTEN functions as a phosphatase that dephosphorylates AKT, thereby reducing its activation and blocking all downstream signaling events. In this study, based on RNA-seq results showing that TAE significantly suppressed AGEs-induced PTEN expression in HK2 cells, we found that sh-*PTEN* markedly reversed the anti-senescence effects of TAE, as evidenced by an increase in SA-β-Gal-positive area and upregulation of senescence-associated proteins. In conclusion, these findings demonstrate that TAE rescues epithelial senescence in DKD by regulating the PTEN/AKT/mTOR pathway. However, its specific active metabolites require further screening and clarification. To this end, a literature review revealed that the main components of TAE, like caffeic acid (1) [14, 15], hesperidin (2) [1, 16], and isoquercitrin (4) [13, 35], all exhibit renoprotective effects. In subsequent studies, we aim to further identify anti-aging substances in TAE and investigate their effects on the PTEN/AKT/mTOR pathway.

However, it should be acknowledged that the direct administration of TAE in this in vitro model cannot fully replicate the complex in vivo metabolic processes and systemic interactions to oral administration. Building on these findings, our future research will systematically isolate the bioactive constituents of TAE and employ drug containing serum to better understand the pharmacokinetic and pharmacodynamic effects of these compounds.

## Conclusion

Taken together, our data firstly demonstrate that the abnormal activation of the mTOR signaling pathway accelerates DKD progression through promoting epithelial senescence and reveal that TAE improves epithelial senescence in DKD by regulating the PTEN/AKT/mTOR pathway. These findings provide novel insights into the mechanisms underlying DKD pathogenesis and highlight TAE as a promising therapeutic agent for DKD.

## Supplementary Information

Supplementary Material 1.Fig S1.** A**–**D** Water intake and food intake during the administration period. **E-H **The urinary ACR (**E**), blood urea nitrogen (**F**), serum creatinine (**G**), and urinary β2-MG (**H**) levels were measured using commercial kits (*n* = 6 per group).** I **Masson staining of kidney tissues. Scale bar = 100 μm. Data are presented as the means ± SEM. ^###^*P* < 0.001 *vs.* Control group; ^*P* < 0.05 *vs.* DKD group. Fig S2.** A** Cell aiablity of HK2 after TAE treatment. **B** mRNA level of PTEN. **C** Protein level detected by Western blot analysis (*n* = 3 per group)

Supplementary Material 2.

## Data Availability

No datasets were generated or analysed during the current study.

## Abbreviations

TAE: *T. triquetrum* aqueous extract
DKD: Diabetic kidney disease
SA-β-Gal: Senescence-associated β-galactosidase
eGFR: Estimated glomerular filtration rate
ESRD: End-stage renal disease
SGLT2: Sodium-glucose cotransporter protein 2
RAAS: Renin-angiotensin-aldosterone system
SASP: Senescence-associated secretory phenotype
TEC: Tubular epithelial cell
DAPA: Dapagliflozin
FBG: Fasting blood glucose
GSP: Glycated serum protein
BUN: Blood urea nitrogen
β2-MG: β2-microglobulin
PAS: Periodic Acid-Schiff
IHC: Immunohistochemistry
α-SMA: alpha-smooth muscle actin
AGEs: Advanced glycation end-products
HK2: Human kidney 2 cells
GO: Gene Ontology
KEGG: Kyoto Encyclopedia of Genes and Genomes
RT-qPCR: Real-time PCR
PCA: Principal component analysis
DEGs: Differentially expressed genes
