# Supplementary material for: Tadehagi triquetrum aqueous extract ameliorates diabetic kidney disease through mitigating epithelial senescence via the PTEN/AKT/mTOR signaling pathway
Source: Chin Med. 2026 Mar 31;21:107. doi: 10.1186/s13020-026-01378-0 (PMC13036931; doi:10.1186/s13020-026-01378-0)
Supplement: Supplementary file 1 — Supplementary Material 1.Fig S1. A–D Water intake and food intake during the administration period. E-H The urinary ACR (E), blood urea nitrogen (F), serum creatinine (G), and urinary β2-MG (H) levels were measured using commercial kits (n = 6 per group). I Masson staining of kidney tissues. Scale bar = 100 μm. Data are presented as the means ± SEM. ###P < 0.001 vs. Control group; ^P < 0.05 vs. DKD group. Fig S2. A Cell aiablity of HK2 after TAE treatment. B mRNA level of PTEN. C Protein level detected by Western blot analysis (n = 3 per group) [file 13020_2026_1378_MOESM1_ESM.docx]

**Supplementary materials**

**
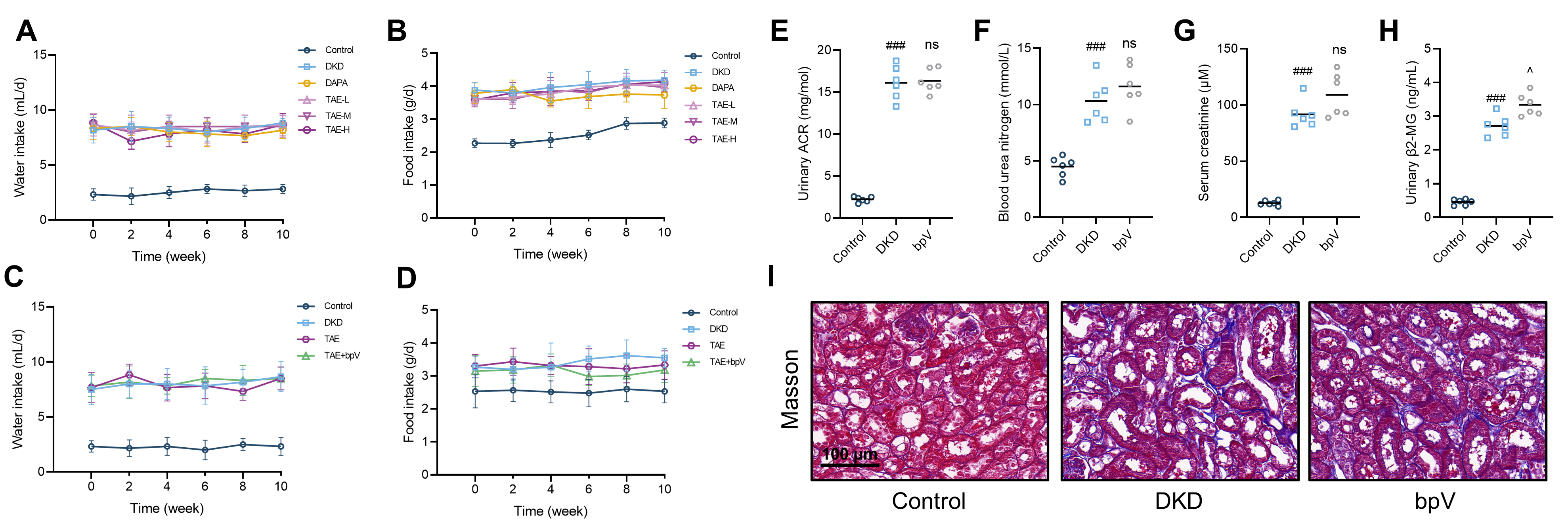
**

**Fig S1. (A-D)** Water intake and food intake during the administration period. **(E-H)** The urinary ACR (E), blood urea nitrogen (F), serum creatinine (G), and urinary β2-MG (H) levels were measured using commercial kits (*n* = 5 per group). **(I)** Masson staining of kidney tissues. Scale bar = 100 μm. Data are presented as the means ± SEM. ^###^*P* < 0.001 *vs.* Control group; ^*P* < 0.05 *vs.* DKD group.

**
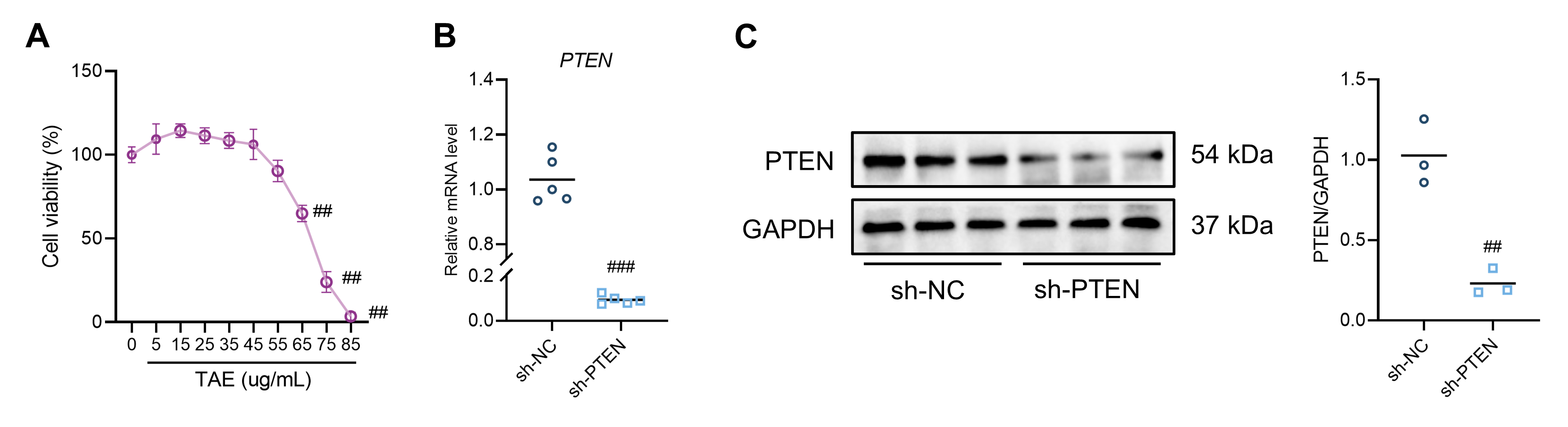
**

**Fig S2. (A)** Cell aiablity of HK2 after TAE treatment. **(B)** mRNA level of PTEN. **(C)** Protein level detected by Western blot analysis (*n* = 3 per group)
