# Supplementary material for: Tadehagi triquetrum aqueous extract ameliorates diabetic kidney disease through mitigating epithelial senescence via the PTEN/AKT/mTOR signaling pathway
Source: Chin Med. 2026 Mar 31;21:107. doi: 10.1186/s13020-026-01378-0 (PMC13036931; doi:10.1186/s13020-026-01378-0)
Supplement: Supplementary file 2 — Supplementary Material 2. [file 13020_2026_1378_MOESM2_ESM.docx]

**Table. S1. Antibodies for Western blot**

| **Antibody** | **Cat No.** | | **Company** | **Dilutions** | **Source** |
| --- | --- | --- | --- | --- | --- |
| Collagen Ⅰ | | 72026T | CST | 1:500 | Rabbit |
| TGF-β | | 49728T | CST | 1:1000 | Rabbit |
| α-SMA | | 14395-1-AP | Proteintech | 1:1000 | Rabbit |
| P53 | | 10442-1-AP | Proteintech | 1:1000 | Rabbit |
| P21 | | 10355-1-AP | Proteintech | 1:1000 | Rabbit |
| P16 | | 10883-1-AP | Proteintech | 1:1000 | Rabbit |
| PI3K | | 20584-1-AP | Proteintech | 1:1000 | Rabbit |
| p-PI3K | | 4228T | CST | 1:500 | Mouse |
| p-AKT(Ser473) | | 66444-1-lg | Proteintech | 1:1000 | Mouse |
| AKT | | 10176-2-AP | Proteintech | 1:1000 | Rabbit |
| p-mTOR(Ser2448) | | 67778-1-lg | Proteintech | 1:1000 | Mouse |
| mTOR | | 20657-1-AP | Proteintech | 1:1000 | Rabbit |
| PTEN | | 60300-3-lg | Proteintech | 1:1000 | Mouse |
| GAPDH | | 10494-1-AP | Proteintech | 1:5000 | Rabbit |
| HRP-Goat Anti-Rabbit Antibody (H+L) | | RGAR001 | Proteintech | 1:2000 |  |
| HRP-Goat Anti-Mouse Antibody (H+L) | | RGAM001 | Proteintech | 1:2000 |  |
